# Aberrant DNA Methylation Predicts Melanoma-Specific Survival in Patients with Acral Melanoma

**DOI:** 10.3390/cancers11122031

**Published:** 2019-12-16

**Authors:** Dinesh Pradhan, George Jour, Denái Milton, Varshini Vasudevaraja, Michael T. Tetzlaff, Priyadharsini Nagarajan, Jonathan L. Curry, Doina Ivan, Lihong Long, Yingwen Ding, Ravesanker Ezhilarasan, Erik P. Sulman, Adi Diab, Wen-Jen Hwu, Victor G. Prieto, Carlos Antonio Torres-Cabala, Phyu P. Aung

**Affiliations:** 1 Department of Pathology, Section of Dermatopathology, The University of Texas MD Anderson Cancer Center, Houston, TX 77030, USA; drdineshpradhan@gmail.com (D.P.); mtetzlaff@mdanderson.org (M.T.T.); pnagarajan@mdanderson.org (P.N.); jlcurry@mdanderson.org (J.L.C.); dsivan@mdanderson.org (D.I.); vprieto@mdanderson.org (V.G.P.); 2 Department of Pathology and Dermatology, NYU Langone Medical Center, New York, NY 10016, USA; george.jour@nyulangone.org (G.J.); varshini.vasudevaraja@nyulangone.org (V.V.); 3 Department of Biostatistics, The University of Texas MD Anderson Cancer Center, Houston, TX 77030, USA; drmilton@mdanderson.org; 4 Department of Translational and Molecular Pathology, The University of Texas MD Anderson Cancer Center, Houston, TX 77030, USA; 5 Department of Dermatology, The University of Texas MD Anderson Cancer Center, Houston, TX 77030, USA; 6 Department of Radiation Oncology, The University of Texas MD Anderson Cancer Center, Houston, TX 77030, USA; llong@mdanderson.org; 7 Department of Radiation Oncology, NYU Langone School of Medicine, New York, NY 10016, USA; yingwen.ding@nyulangone.org (Y.D.); ravesanker.ezhilarasan@nyulangone.org (R.E.); erik.sulman@nyulangone.org (E.P.S.); 8 Department of Melanoma Oncology, The University of Texas MD Anderson Cancer Center, Houston, TX 77030, USA; adiab@mdanderson.org (A.D.); hwu.wenjen@gmail.com (W.-J.H.)

**Keywords:** epigenetics, methylation, acral lentiginous melanoma, cutaneous melanoma

## Abstract

Acral melanoma (AM) is a rare, aggressive type of cutaneous melanoma (CM) with a distinct genetic profile. We aimed to identify a methylome signature distinguishing primary acral lentiginous melanoma (PALM) from primary non-lentiginous AM (NALM), metastatic ALM (MALM), primary non-acral CM (PCM), and acral nevus (AN). A total of 22 PALM, nine NALM, 10 MALM, nine PCM, and three AN were subjected to genome-wide methylation analysis using the Illumina Infinium Methylation EPIC array interrogating 866,562 CpG sites. A prominent finding was that the methylation profiles of PALM and NALM were distinct. Four of the genes most differentially methylated between PALM and NALM or MALM were *HHEX*, *DIPK2A*, *NELFB*, and *TEF*. However, when primary AMs (PALM + NALM) were compared with MALM, *IFITM1* and *SIK3* were the most differentially methylated, highlighting their pivotal role in the metastatic potential of AMs. Patients with NALM had significantly worse disease-specific survival (DSS) than patients with PALM. Aberrant methylation was significantly associated with aggressive clinicopathologic parameters and worse DSS. Our study emphasizes the importance of distinguishing the two epigenetically distinct subtypes of AM. We also identified novel epigenetic prognostic biomarkers that may serve to risk-stratify patients with AM and may be leveraged for the development of targeted therapies.

## 1. Introduction

Primary cutaneous melanomas (CMs) arising from the glabrous skin of the palms, soles, or nail apparatus are considered acral melanomas (AMs). It has been suggested that AMs are associated with a worse prognosis than other CMs, mainly because of advanced stage at presentation, which may be related to the relatively occult anatomic sites and inconspicuous appearance [1,2,3,4,5,6]. Most AMs exhibit a lentiginous proliferation within the epidermis and are referred to as acral lentiginous melanoma (ALM), a rare subtype characterized by increased risk of metastasis and melanoma-specific death [1,2,3]. ALM is the most common melanoma subtype in African American, Hispanic, and Asian individuals, accounting for 29% to 72% of CM cases in these populations [7,8,9]. Genomic analyses of CM have yielded biological and therapeutic insights, but understanding of the molecular pathogenesis of AM remains limited [10,11,12,13]. Whereas melanoma on sun-exposed sites harbors a large number of ultraviolet light–induced mutations commonly affecting genes regulating the MAPK pathway, it has been reported that AM is driven by a combination of amplifications of *TERT*, *CCND1*, *CDK4*, *MITF*, *PAK1*, *GAB2*, *YAP1*, and *MDM2* and mutations in *BRAF*, *NRAS*, *KIT*, and *PDGFRA* [10,11,12,13].

Aberrant DNA methylation is a frequent epigenetic change in melanoma and has prognostic implications [14]. Epigenetic changes are more frequent than genetic alterations in melanoma and are potentially reversible [15,16,17,18]. Recent studies have revealed that promoter methylation of *PTEN* is an independent predictor of worse survival in melanoma [19,20]. Methylation of *RARB*, *APC*, *CDH13*, *ESR1*, *CDKN2A*, *RASSF1*, *MGMT*, and *HOXD9* has been shown to be associated with poor survival in CM [21,22,23,24,25,26]. AMs have relatively greater prevalence of DNA methylation than other types of CM and are significantly associated with *PTEN* and *CDH13* hypermethylation [21]. Genome-wide mapping of 5-hydroxymethylcytosine revealed its loss in melanoma, and restoring active *TET2* or *IDH2* reactivated 5-hydroxymethylcytosine suppressed melanoma growth and increased tumor-free survival in animal models, suggesting the therapeutic potential of targeting epigenetic changes [27,28].

Despite promising clinical responses to immune checkpoint blockade therapy and targeted therapy, the response of ALM to these agents remains unpredictable, underscoring a critical need to delineate additional prognostic and predictive biomarkers and/or novel therapeutic targets for this disease. To date, few studies have focused on identifying epigenetic prognostic biomarkers in AM [21]. Here, we subclassified AMs into primary ALM (PALM), non-ALM-type melanomas involving acral skin (NALM, defined as AM that lacks lentiginous pattern of intraepidermal melanocytic growth; includes superficial spreading, lentigo maligna, and nodular types), and metastatic ALM (MALM), and interrogated the methylation profiles of AMs and primary non-acral CM (PCM). We aimed to compare the methylome of PALM and NALM as they are histologically distinct as well as primary AM and MALM, and determine the association of these methylome signatures with clinicopathologic features, overall survival (OS), and disease-specific survival (DSS).

## 2. Results

### 2.1. Clinicopathologic Features

Most patients in our cohort were white men with intermediate or thick melanomas. The key clinicopathologic features are summarized in Table 1.

### 2.2. Association of Melanoma Subtype with Clinicopathologic Parameters, Genetic Alterations, and Outcome

There were no significant differences among melanoma subtypes in sex, age, race, American Joint Committee on Cancer (AJCC) stage, Clark level, Breslow thickness, mitotic rate, ulceration, regression, perineural invasion, microsatellitosis, or genetic alteration (Table 1). Of all the histopathologic parameters examined, only perineural invasion was associated with worse survival (OS, *p* = 0.014; DSS, *p* = 0.019).

**Figure 1:**
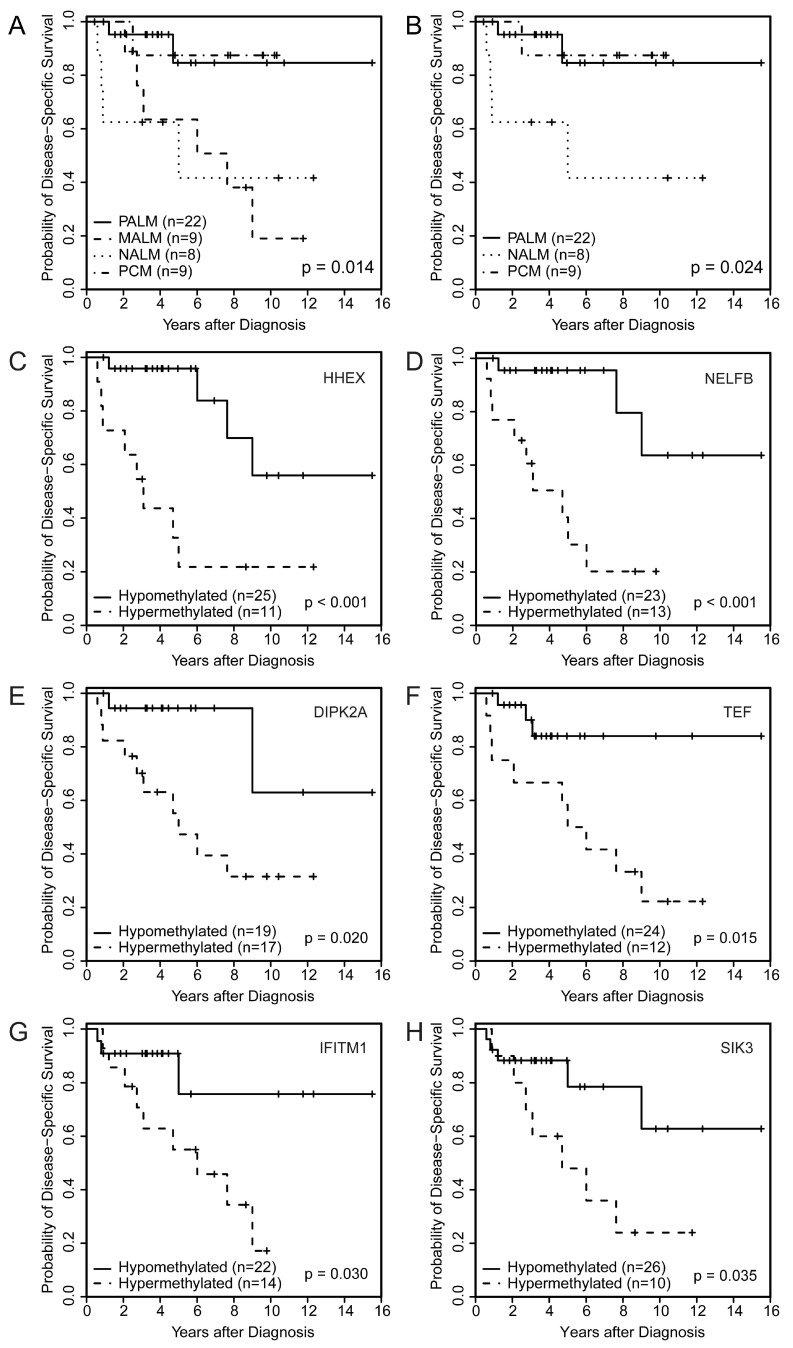
Disease-specific survival by melanoma subtype among (**A**) the four subtypes (*p* = 0.014) and (**B**) three subtypes (*p* = 0.024); (**C**–**H**) Disease-specific survival by specific methylome probes showing significant correlation between aberrant methylation of (**A**) *HHEX*, (**B**) *NELFB*, (**C**) *DIPK2A*, (**D**) *TEF*, (**E**) *IFITM1*, and (**F**) *SIK3* and worse disease-specific survival.

Although our sample size was too small to have statistical power for making a definite statement, patients with NALM (median: 5.0 years) had significantly worse DSS than patients with PALM (median not reached), MALM (median: 7.6 years), or PCM (median not reached) (Figure 1A,B) (*p* = 0.02 among all four groups [PALM, MALM, NALM, and PCM] and *p* = 0.024 among PALM, NALM, and PCM).

### 2.3. Performance of the Methylome Signature in Distinguishing Malignant from Benign Melanocytic Neoplasms

The significant promoter-associated differentially methylated positions for all groups are summarized in Supplementary Table S1. These positions constituted the methylome signature.

To ensure that the algorithm functioned adequately, we tested its performance in distinguishing malignant from benign acral melanocytic neoplasms. All cases of NALM and PCM were analyzed in parallel with AN. As expected, melanoma cases clustered tightly together and were clearly separated from AN cases (Supplementary Figure S1A,B). NALM and PCM showed significant enrichment of the MAPK pathway, which seemed to be a more common epigenetic mechanism in these melanomas than in AN (Supplementary Figure S2A; Supplementary Table S2).

### 2.4. Identification of a Primary Acral Lentiginous Melanoma (PALM)-Specific Methylome Signature

In order to identify a PALM-specific methylome signature, we compared the methylation profiles of PALM versus NALM and PALM versus MALM. The top 5000, 1000, 500, and 100 differentially methylated probes (DMPs) (the probes with the lowest associated *p* and *q* values) were selected (Supplementary Table S2). Given the high correlations among all four sets of probes (*R*^2^ = 0.98), we present the heatmap based on the top 50 DMPs.

**Figure 2:**
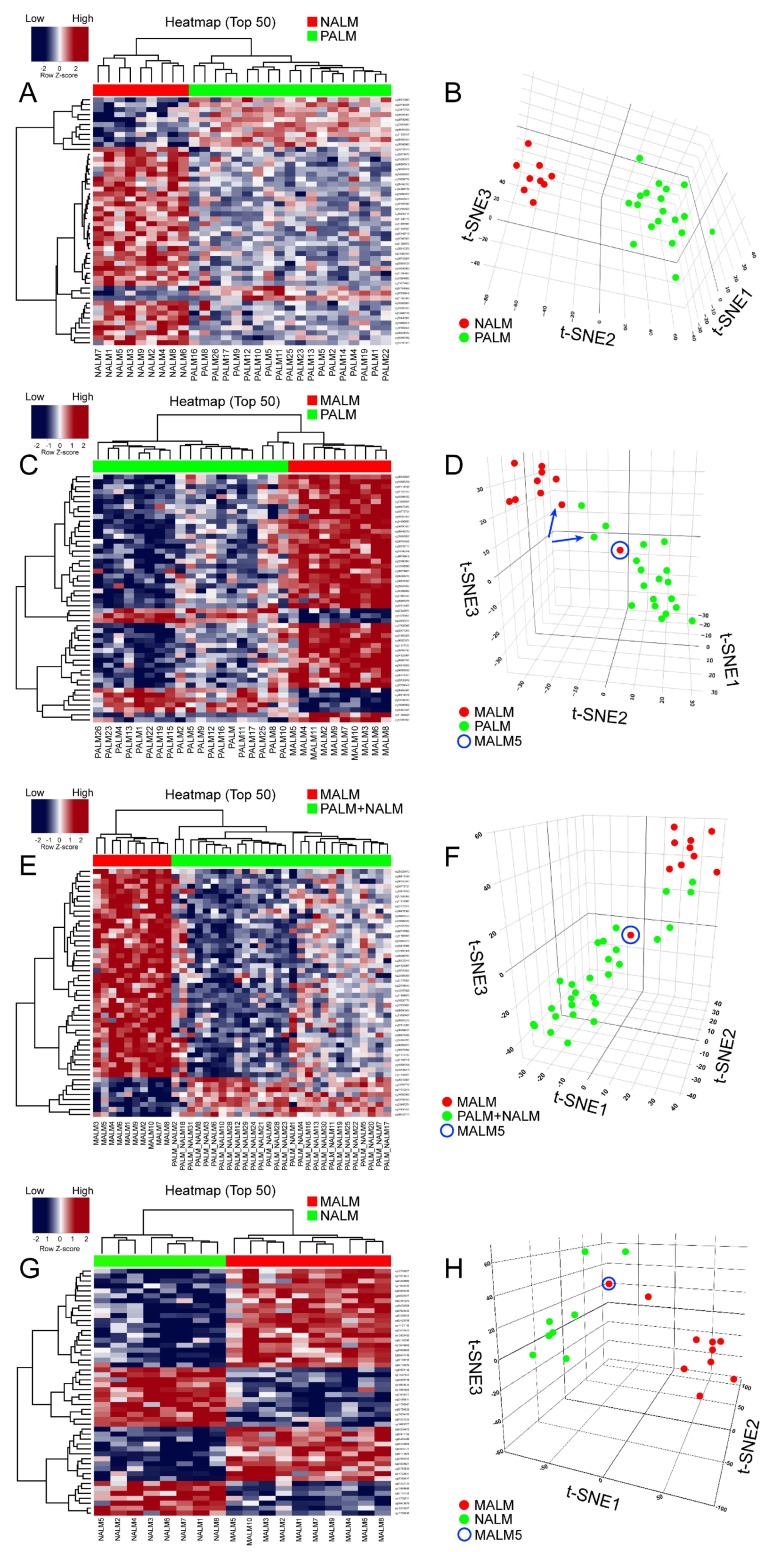
Results of methylation analyses. (**A**,**C**,**E**,**G**): Unsupervised hierarchical clustering heatmaps with different β-scores for the 50 most significantly differentially methylated gene-coding and non-gene-coding CpG islands (i.e., lowest *p* and *q* values [*p* < 0.05; *q* < 0.01]). The heatmap shows distinct methylation profiles between (**A**) PALM and NALM, (**C**) PALM and MALM, (**E**) PALM + NALM and MALM and (**G**) NALM and MALM. Loci hypermethylated in one tumor are hypomethylated in the other and vice versa. (In the heatmap: RED corresponds to hypermethylation or low gene expression and BLUE corresponds to hypomethylation or high gene expression) (**B**,**D**,**F**,**H**): Three-dimensional T-distributed stochastic neighbor embedding (t-SNE) showing the distribution of cases classified on the basis of differentially methylated probes adjusted for age and sex. The algorithm calculates the similarity of the patient samples in the two compared groups in a 3-dimensional space, in this case labeled as t-SNE1; t-SNE2; and t-SNE3. The numbers in the three different axes do not have units; they represent the approximate distance between the two different groups/clusters and reflect whether they are truly distinct or not. (**B**) NALM (red dots) and PALM (green dots) show no neighboring and discrete clusters. (**D**) MALM (red dots) and PALM (green dots) showing discrete clusters. Note that case MALM 5 (circle) is an outlier that clusters with the PALM group. The blue arrows indicate paired samples from the same patient. (**F**) MALM (red dots) and PALM + NALM (green dots). Note that case MALM5 (circle) is an outlier that clusters with the PALM + NALM group. (**H**) NALM (green dots) and MALM (red dots) show discrete clusters with the exception of MALM5 (circle) that clusters with the NALM group.

Unsupervised hierarchical clustering and multidimensional parametric analysis showed different methylation profiles and distinct clusters in the PALM and NALM groups (Figure 2A,B; Supplementary Table S2). Four probes that were most differentially methylated between PALM and NALM co-localized to the promoter region of genes of interest: cg26732804 (*HHEX*), cg14397361 (*NELFB* [formerly *COBRA1*]), cg21298070 (*TEF*), and cg07088959 (*DIPK2A* [formerly *C3orf58*]). All four probes exhibited significant differences in methylation status between PALM and NALM with profound hypomethylation in PALM (specific identified cutoffs <0.024, <0.014, <0.0434, and <0.200, respectively; *p* = 0.0001, *p* = 0.047, *p* = 0.0014, and *p* = 0.0034, respectively) (Supplementary Table S3). As expected, pathway network analysis revealed enrichment in 233 genes pertaining to the MAPK signaling pathway (Supplementary Figure S2B; Supplementary Table S1).

When we compared PALM and MALM, we found that the four promoter-associated probes above-mentioned were among the most differentially methylated (Figure 2C,D; Supplementary Table S1). Three of the four probes exhibited significant differences in methylation status between PALM and MALM with profound hypomethylation (using the above-mentioned cutoffs) in PALM (*HHEX*, *p* = 0.04; *DIPK2A*, *p* = 0.02; *NELFB*, *p* = 0.002); there was no significant methylation difference in *TEF* (*p* = 0.11) (Supplementary Table S3). Notably, cg26732804 (*HHEX*) showed a greater range of hypomethylation than the other three probes, with hypomethylation in 14 (73%) of 19 PALM cases versus three (30%) of 10 MALM cases. Pathway network analysis revealed enrichment in 273 genes pertaining to the HPV signaling pathway (Supplementary Figure S3A; Supplementary Table S1). Notably, when we compared NALM and MALM, the cases in each subgroup showed discrete clustering with the exception of MALM5 that clustered with the NALM group (Figure 2G,H).

### 2.5. Association of PALM-Specific Methylome Signature with Clinicopathologic Parameters and Outcome

Univariate Cox proportional hazards regression models showed that hypermethylation of *HHEX* and *NELFB* significantly correlated with poor OS (*HHEX*: hazard ratio [HR], 4.60; 95% CI, 1.63 to 13.00; *p* = 0.004; *NELFB*: HR, 4.75; 95% CI, 1.61 to 13.99; *p* = 0.005) and poor DSS (*HHEX*: HR, 6.13; 95% CI, 1.83 to 20.49; *p* = 0.003; *NELFB*: HR, 7.17; 95% CI, 1.92 to 26.76; *p* = 0.003). Kaplan–Meier survival analysis also demonstrated that hypermethylation of *HHEX* and *NELFB* significantly correlated with poor OS (Supplementary Figure S4A,B) and poor DSS (Figure 1C,D). Hypermethylation of cg26732804 (*HHEX*) and cg14397361 (*NELFB*) was significantly associated with the presence of lymph node metastasis (odds ratio [OR], 7.50; 95% CI, 1.61 to 34.95; *p* = 0.01 and OR, 5.50; 95% CI, 1.16 to 26.14; *p* = 0.032, respectively). These findings corroborated the prognostic impact of hypermethylation of these loci on survival (Table 2). Another probe identified among the top 50 DMPs was cg02753722 (*CDH13)*. Using the identified cutoff (<0.226), hypermethylation of cg02753722 (*CDH13*) was associated with worse OS and DSS among patients with AM (PALM + NALM) (*p* = 0.015 and *p* = 0.0009, respectively). cg21298070 (*TEF*) hypermethylation was associated with a higher mitotic rate (>10) (OR, 14.44; 95% CI, 1.56 to 133.6; *p* = 0.019).

Aberrant methylation was significantly associated with aggressive clinicopathologic parameters including the presence of lymph node metastasis, higher Breslow thickness, increased mitoses, ulceration, and perineural invasion (Table 2). Kaplan–Meier survival analysis demonstrated significant correlations between the hypomethylation of *CDH13* and worse OS and DSS (Supplementary Figure S4C,D) and between the hypermethylation of *DIPK2A* (Figure 1E) and *TEF* (Figure 1F) and worse DSS (Table 2).

### 2.6. Identification of a Specific Methylome Signature Associated with Metastasis from Acral Melanomas (AMs)

**Figure 3:**
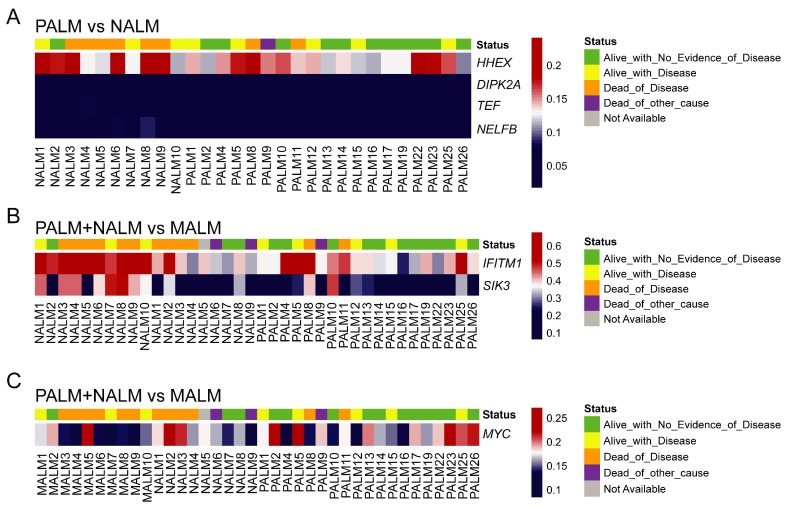
Prevalence and degree of hypomethylation (using the raw β score) of the probes of interest across the studied samples. (**A**) Significant probes associated with PALM vs. NALM: cg26732804 (*HHEX*), cg07088959 (*DIPK2A*), cg21298070 (*TEF*), and cg14397361 (*NELFB).* (**B**) Significant probes associated with metastasis of AM: cg11694510 (*IFITM1)* and cg09923443 (*SIK3)*. (**C**) Significant probes associated with metastasis of AM: cg24666276 (*MYC)*. In each panel, each column represents one sample; the top row indicates the clinical outcome of the patient that the sample came from; and each row indicates the degree of hypomethylation of the corresponding probe/gene in that sample.

Primary AMs have a significantly better survival than MALM [1,12]. It is important to find prognostic biomarkers that may serve to risk-stratify patients with AM at an early stage of the disease. Comparison of both groups of primary AM (PALM and NALM) to their metastatic counterparts (MALM) showed distinct clusters corresponding to primary and metastatic tumors (Figure 2E,F; Supplementary Table S1). Pathway network analysis revealed enrichment in 233 genes pertaining to the MAPK signaling pathway (Supplementary Figure S3B; Supplementary Table S2). We identified 25 shared probes within the top 50 DMPs in the PALM versus MALM and PALM + NALM versus MALM analyses (Supplementary Figure S5). Within this 25-probe set, two probes, cg11694510 and cg09923443, localized to the promoter regions of *IFITM1* and *SIK3,* respectively. These probes exhibited significantly different levels of methylation between primary and metastatic AM, with profound hypomethylation in the primary tumors (*SIK3*, *p* < 0.0001; *IFITM1*, *p* < 0.0001), highlighting their pivotal role in the initiation of metastasis. Additionally, there was a statistically significant difference (*p* = 0.03) in the methylation status of the *MYC* promoter (probe cg24666276) between primary and metastatic cases with a tendency to hypomethylation in the metastatic cases, suggesting overexpression of MYC and corroborating its role in metastasis. Probe cg11694510 (*IFITM1*) did not show evidence of hypomethylation in any of the MALM cases, but was profoundly hypomethylated in six of 28 (21%) primary (PALM + NALM) cases. Similarly, cg09923443 (*SIK3*) was hypomethylated in only two of 10 (20%) MALM cases, but in 22 of 28 (78%) PALM + NALM cases (Figure 3).

Interestingly, methylation analysis of paired samples of primary tumor (PALM25) and metastatic tumor (MALM10) from the same patient demonstrated that their methylation profiles clustered with those of the corresponding primary and metastatic ALM groups, respectively (Figure 2C,D), revealing distinct methylation profiles.

Although the sample size was small, hypermethylation of IFITM1 was significantly more common in AMs lacking BRAF V600E mutations than in BRAF V600E–mutant AMs (*p* = 0.018).

### 2.7. Association of Aberrantly Methylated Genes in Metastatic AM with Clinicopathologic Parameters and Outcome

Hypermethylation of *IFITM1* and *SIK3* significantly correlated with worse DSS by univariate Cox proportional hazards regression analysis (*IFITM1*: HR, 3.88; 95% CI, 1.04 to 14.48; *p* = 0.044; *SIK3*: HR, 3.26; 95% CI, 1.02 to 10.39; *p* = 0.046) and Kaplan–Meier survival analysis (Figure 1G,H). Additionally, we noted a significant association of hypermethylation of cg09923443 (*SIK3*) and adverse histologic parameters in primary melanomas including ulceration and increased mitotic rate (Table 2). This finding corroborated the potential impact of these two genes on primary tumor aggressiveness and early development of metastasis.

## 3. Discussion

Here, we demonstrate the epigenetic differences among melanomas occurring on acral sites but displaying different histopathological features. The four genes that were the most differentially methylated between PALM and NALM were *HHEX*, *NELFB/COBRA1*, *TEF*, and *DIPK2A/C3orf58*. Our findings suggest profound hypomethylation of the promoters of those genes in PALM compared to NALM. This finding is in line with the worse OS in patients who had hypermethylation of *HHEX* and *NELFB*.

*HHEX* (hematopoietically expressed homeobox) encodes a member of the homeobox family of transcription factors, many of which are involved in development, lymphangiogenesis, and hematopoietic stem cell differentiation [29,30]. HHEX is a transcriptional regulator of the VEGFC/FLT4/PROX1 signaling axis involved in vascular development [29]. HHEX was found to play a role in the migration and invasion of breast and prostate epithelial cells through the direct transcriptional regulation of *Endoglin*. The association of *HHEX* with hematopoietic, colorectal, liver, breast, prostate, and thyroid cancers has been extensively reported [31,32,33,34,35,36,37,38].

*NELFB/COBRA1* (negative elongation factor/co-factor of BRCA1) encodes an essential component of the NELF complex, which negatively regulates the elongation of transcription by RNA polymerase II. The NELF complex acts in association with the DSIF [5,6-dichloro-1-β-D-ribofuranosylbenzimidazole (DRB) sensitivity-inducing factor] complex, causing transcriptional pausing [39]. Thus, hypermethylation of *NELFB*, which is believed to lead to global gene silencing, may result in cellular proliferation and promotion of tumorigenesis. This could explain, at least in part, the worse OS noted in the NALM group compared to the PALM group. *NELFB* appears to play an important role in other tumors including breast cancer, and its mRNA was found to be increased in breast cancer cell lines [40]. An association of *NELFB* with prostate cancer and upper gastrointestinal adenocarcinomas, with overexpression of *NELFB* mRNA in these tumors, has been reported [39,41].

*TEF* (transcriptional enhancer factor) encodes a member of the PAR (proline and acidic amino acid-rich) subfamily of bZIP (basic region/leucine zipper) transcription factors. The TEF/TEAD family of transcription factors are major mediators of YAP (Yes-associated protein) transcriptional activity. YAP and its paralog protein TAZ (transcriptional coactivator with PDZ-binding motif) are downstream effectors of the Hippo pathway and have been shown to facilitate the invasiveness and metastatic potential of melanoma cells [42,43]. Recently, *YAP1* was found to be co-amplified with *PAK1* and *GAB2* on targeted genomic profiling of AM, which is in agreement with our finding of hypomethylation of the promoter of *TEF* [12].

*DIPK2A* (divergent protein kinase domain 2A) encodes a protein-coding gene that plays a role in cardiomyocyte proliferation through paracrine signaling and activation of the PI3K-AKT-CDK7 signaling cascade [44]. An association of *DIPK2A* with cancer has not been reported so far.

While PALM and NALM are epigenetically distinct, our data suggest that AMs, whether of PALM or NALM subtype, share a methylation signature and that the mechanism underlying metastasis is independent of the AM subtype (PALM or NALM). Among the top 50 DMPs, two probes, cg11694510 and cg09923443, localizing to the promoter regions of *IFITM1* and *SIK3*, respectively, showed profound hypomethylation in the primary tumors compared to the metastatic tumors, highlighting the role of these genes in early stromal invasion and initiation of metastasis.

*IFITM1* (interferon-induced transmembrane protein (1) is one of the interferon-stimulated genes that is STAT2 (signal transducer and activator of transcription (2) dependent, and its overexpression augments the proliferation, migration, and invasion of inflammatory breast cancer cells [45]. *IFITM1* expression plays an important role in the invasion and progression of early-stage head and neck cancer and is overexpressed in these tumors [46]; it also promotes metastasis of colorectal cancer [47]. However, a role for *IFITM1* in melanoma has never been reported. Several studies have shown the therapeutic potential of *IFITM1* regulation in breast and cervical squamous cell carcinoma [48,49]. This provides a rationale to investigate the therapeutic potential of agents targeting the regulation of this gene.

*SIK3* (salt-inducible kinase-3) encodes a salt-sensitive kinase that plays important roles in physiological functions including cell proliferation, apoptosis, and survival. *SIK3* expression is increased in the presence of salt and interleukin-17, leading to breast cancer cell proliferation. *SIK3* expression also increases the expression of chemokine CXCL12 and its specific receptor CXCR4 on cancer cells, promoting metastasis [50]. *SIK3* is known to be associated with breast, hepatocellular, and ovarian cancer [51]. It has also been reported to be involved in melanogenesis through the cAMP-response element binding protein and its activator LKB1 (liver kinase B1) [52]. *STK11* (also known as *LKB1* and *PAR-4*) encodes a serine/threonine kinase found in approximately 10% of melanomas [53]. Interestingly, when all AM cases (PALM, NALM, and MALM) were analyzed together, hypermethylation of *IFITM1* and *SIK3* was associated with worse OS. This finding corroborated the potential role of these genes in early stromal invasion and initiation of metastasis.

In agreement with previous studies on CM, we identified significant associations between aberrant methylation and aggressive clinicopathologic parameters in AM including the presence of lymph node metastasis, ulceration, increased mitoses, and higher Breslow thickness [21,22,23,24,25,26]. In addition, our study revealed significant correlation between aberrant methylation of *HHEX*, *DIPK2A*, *NELFB/COBRA1*, *CDH13*, *TEF*, *IFITM1*, and *SIK3* and worse DSS.

A recent study interrogating AMs by targeted deep sequencing identified two distinct subtypes of melanoma on acral sites: the *BRAF* mutant, similar to nonacral melanoma, and non-*BRAF* mutant [12]. Interestingly, in our small cohort, we observed that the hypermethylation of IFITM1 was significantly more common in *BRAF* V600E negative AMs than in *BRAF* V600E-mutant AMs. Although validation by larger studies is warranted, it could be hypothesized that these distinct genetic subtypes of AMs harbor distinct methylation profiles.

There are some limitations in our study:(1) Relatively small sample size; (2) retrospective study; (3) in our cases, conventional prognostic indicators did not reveal statistical significance; (4) treatment regimens and surgical management have not been considered in survival analysis; and (5) we did not confirm the differential expression of methylated (hypo- versus hyper-) genes in AMs, although the project is under consideration.

Regardless, the epigenetic biomarkers identified in our study serve as a rationale to further validate these findings on larger independent cohorts. Once validated, these findings would serve to risk-stratify AM patients at their early disease stage (primary tumors) for more aggressive adjuvant treatment regimens. They may serve as potential therapeutic targets with methylation modulators for personalized management of patients with these tumors.

## 4. Materials and Methods

### 4.1. Patient Cohort

After receiving institutional review board approval (PA12-0494), we reviewed our pathology database and identified all cases of PALM, NALM, MALM, PCM, and AN diagnosed at our institution during 2002–2018. We excluded cases for which either tissue was not available in our institutional repository or the amount of DNA extracted from the tumor was not sufficient for methylation studies, generating the following final cohort for our study: 22 PALM, nine NALM, 10 MALM, nine PCM, and three AN. All slides for each case were reviewed by two pathologists (DP and PPA) to confirm the diagnosis and select the most viable tumor areas for DNA extraction. The clinicopathologic features of one case of NALM were not available, and this case was excluded from the analyses of survival and histologic parameters. We also had paired samples of MALM and PALM from one patient, and only the PALM sample was included in the survival and histologic analyses.

### 4.2. DNA Isolation, Methylation Analysis, Functional Genomic Pathway Analysis, and Statistical Analysis

DNA isolation, methylation analysis, and functional genomic pathway analysis were performed as described in our previous publication [54] (please see details in the Supplementary Methods in Supplementary Materials, available online). Briefly, DNA extraction (Thermo Fisher) followed by bisulfite conversion (Zymo Research, Irvine, CA, USA) was performed as per the manufacturer’s instructions. The Infinium Methylation EPIC array (Illumina, San Diego, CA, USA) was used to determine the DNA methylation status of 866,562 CpG sites, following the manufacturer’s instructions. The DMPs finder function in minfi was used to identify DMPs for three different comparisons: (1) PALM versus NALM, (2) PALM versus MALM (3), and AM (PALM + NALM) versus MALM. Three PALMs and four PCMs did not pass the quality-control test for the assay and hence were excluded from the analysis. All statistical analyses were performed using SAS 9.4 for Windows (SAS Institute Inc., Cary, NC, USA).

### 4.3. Data Access

IDAT files of our cohort (48 samples) have been deposited in Gene Expression Omnibus, accession number GSE133395. The R script used for the analysis has been uploaded to Github (https://github.com/varshivasu7/minfi_Illumina_methylation) [54].

## 5. Conclusions

In conclusion, we have identified four novel epigenetic prognostic biomarkers in AM: *HHEX*, *NELFB*, *IFITM1*, and *SIK3*. Hypermethylation in the promoter region of these genes is significantly associated with aggressive clinicopathologic parameters including greater Breslow thickness, ulceration, increased mitotic rate, and lymph node metastasis and worse DSS in AM. Our study also revealed that PALM and NALM are epigenetically distinct subtypes and that NALM is associated with worse DSS; this emphasizes the importance of distinguishing these two types. These are promising findings that, after further validation through larger studies and mechanistic functional studies, can be translated in the clinical setting. Once validated, the epigenetic biomarkers identified in this study can serve to stratify patients for more aggressive treatment regimens. Our study confirms methylation array testing as a robust approach for epigenetic analysis that is resistant to factors that are known to interfere with gene expression analysis including formalin fixation, ischemia time, and others. Finally, our study provides a rationale to investigate agents targeting epigenetic regulation in AM, potentially expanding the limited therapeutic options currently available to patients with this disease.

## Figures and Tables

**Table 1:** Summary of patient and clinical characteristics overall and by melanoma subtype *.

Characteristic	All (N = 48)	PALM (N = 22)	MALM (N = 9) ^†^	NALM (N = 8) ^†^	PCM (N = 9)	*p* Value #
Sex						
Male	30 (63)	14 (64)	5 (56)	5 (63)	6 (67)	0.97
Female	18 (38)	8 (36)	4 (44)	3 (38)	3 (33)	
Age, median (range), y	62.1 (1.7–90.6)	67.4 (36.6–90.6)	58.4 (16.0–79.0)	55.6 (1.7–89.4)	62.3 (13.7–79.0)	0.22
Race						
White	40 (83)	16 (73)	8 (89)	7 (88)	9 (100)	0.34
Other	8 (17)	6 (27)	1 (11)	1 (13)	0	
AJCC 8th edition stage at presentation						
I	10 (21)	6 (27)	1 (13)	1 (13)	2 (22)	0.37
II	15 (32)	7 (32)	1 (13)	2 (25)	5 (56)	
III	21 (45)	9 (41)	6 (75)	4 (50)	2 (22)	
IV	1 (2)	0	0	1 (13)	0	
Site(s) of LN metastasis at diagnosis						
None	25 (54)	13 (59)	3 (33)	1 (17)	8 (89)	**0.019**
Enlarged ^‡^	1 (2)	1 (5)	0	0	0	
Regional	1 (2)	0	1 (11)	0	0	
Sentinel	17 (37)	8 (36)	3 (33)	5 (83)	1 (11)	
Regional and sentinel	2 (4)	0	2 (22)	0	0	
Total number of LNs with metastasis at diagnosis, median (range)	2 (1–11)	2 (1–3)	4 (1–11)	2 (1–2)	1 (1–1)	0.24
Primary histologic subtype						
ALM	31 (65)	22 (100)	9 (100)	0	0	**<0.001**
LMM	2 (4)	0	0	0	2 (22)	
NM	6 (13)	0	0	4 (50)	2 (22)	
SSM	5 (10)	0	0	3 (38)	2 (22)	
Unclassified	4 (8)	0	0	1 (13)	3 (33)	
Clark level						
II	1 (2)	1 (5)	0	0	0	0.79
III	1 (2)	0	0	1 (13)	0	
IV	32 (68)	15 (68)	6 (75)	4 (50)	7 (78)	
V	13 (27)	6 (27)	2 (22)	3 (38)	2 (22)	
Breslow thickness, mm						
1.01–2	13 (28)	7 (33)	2 (25)	2 (25)	2 (22)	0.34
2.01–4	14 (30)	4 (19)	1 (13)	4 (50)	5 (56)	
>4	19 (41)	10 (48)	5 (63)	2 (25)	2 (22)	
Radial growth phase						
Present	25 (74)	13 (87)	6 (100)	2 (33)	4 (57)	**0.021**
Not identified	9 (26)	2 (13)	0	4 (67)	3 (43)	
Vertical growth phase						
Present	46 (100)	22 (100)	8 (100)	7 (100)	9 (100)	
Not identified	0	0	0	0	0	
Mitotic figures						
<1	2 (5)	1 (5)	0	1 (17)	0	0.49
1–4	30 (68)	14 (64)	5 (63)	3 (50)	8 (100)	
5–9	5 (11)	3 (14)	2 (25)	0	0	
10–20	5 (11)	2 (9)	1 (13)	2 (33)	0	
>20	2 (5)	2 (9)	0	0	0	
Ulceration						
Present	21 (48)	11 (52)	6 (75)	2 (29)	2 (25)	0.18
Not identified	23 (52)	10 (48)	2 (25)	5 (71)	6 (75)	
Regression						
Present	6 (14)	4 (18)	1 (13)	0	1 (13)	0.92
Not identified	38 (86)	18 (82)	7 (88)	6 (100)	7 (88)	
Vascular invasion						
Present	6 (13)	2 (9)	1 (11)	3 (50)	0	**0.05**
Not identified	40 (87)	20 (91)	8 (89)	3 (50)	9 (100)	
Perineural invasion						
Present	13 (30)	7 (32)	3 (38)	2 (33)	1 (13)	0.73
Not identified	31 (70)	15 (68)	5 (63)	4 (67)	7 (88)	
Microscopic satellitosis						
Present	4 (9)	2 (9)	0	1 (17)	1 (13)	0.71
Not identified	40 (91)	20 (91)	8 (100)	5 (83)	7 (88)	
TIL						
Non-brisk	45 (100)	22 (100)	8 (100)	7 (100)	8 (100)	
Brisk	0	0	0	0	0	
Associated nevus						
Present	3 (7)	1 (5)	0	0	2 (25)	0.23
Not identified	41 (93)	21 (95)	8 (100)	6 (100)	6 (75)	
Predominant cytology						
Epithelioid	28 (64)	14 (67)	4 (50)	6 (86)	4 (50)	0.22
Nevoid	5 (11)	2 (10)	0	0	3 (38)	
Spindled	11 (25)	5 (24)	4 (50)	1 (14)	1 (13)	
Genetic mutation						
Yes	15 (48)	6 (38)	4 (44)	3 (100)	2 (67)	0.29
No	16 (52)	10 (63)	5 (56)	0	1 (33)	
Vital status						
Alive with NED	20 (42)	12 (55)	1 (11)	2 (25)	5 (56)	**0.006**
Alive with disease	7 (15)	5 (23)	2 (22)	0	0	
Died of other causes	8 (17)	3 (14)	0	2 (25)	3 (33)	
Died with disease	13 (27)	2 (9)	6 (67)	4 (50)	1 (11)	
Follow-up time after diagnosis (all patients), median (range), mo	54.9 (5.2–186.0)	47.7 (11.0–186.0)	72.1 (25.0–141.1)	43.0 (7.1–147.8)	93.7 (5.2–123.9)	0.42
Survival time after diagnosis (survivors) ^§^, months						
Number of patients	27	17	3	2	5	0.07
Median (range)	68.0 (5.2–186.0)	48.9 (18.6–186.0)	103.8 (29.7–141.1)	136.5 (125.1–147.8)	93.7 (57.6–123.9)	

Abbreviations: LMM, lentigo maligna melanoma; MALM, metastatic ALM; NALM, primary non-lentiginous AM involving acral sites; LN, lymph node; NED, no evidence of diseases; NM, nodular melanoma; PALM, primary acral lentiginous melanoma; PCM, primary non-acral cutaneous melanoma; SSM, superficial spreading melanoma; TIL, tumor-infiltrating lymphocytes. * Values in table are number of patients (percentage) unless otherwise indicated. ^†^ The clinicopathologic features of 1 case of NALM were not available, hence clinicopathologic data of 8 NALM cases are provided in this table although methylation of 9 NALM cases was analyzed. Also, we had paired samples of MALM and PALM from the same patient in one case, and only the PALM data were used in the survival and histologic analyses. ^‡^ Enlarged means that the patient had enlarged nodes clinically but nodal metastasis was not confirmed histopathologically. ^§^ For the patients still alive at their last follow-up visit. # Statistically significant *p* values (≤ 0.05) are marked in bold.

**Table 2:** Associations between survival and histologic parameters and β-score optimal cutoff groups (univariate analysis) *.

**Survival**	**Genes**	**HR (95% CI)**	***p* Value**
Overall	*CDH13*	0.29 (0.10 to 0.84)	0.022
	*NELFB*	4.75 (1.61 to 13.99)	0.005
	*HHEX*	4.60 (1.63 to 13.00)	0.004
Disease-specific	*CDH13*	0.22 (0.06 to 0.74)	0.014
	*DIPK2A*	5.09 (1.11 to 23.34)	0.036
	*NELFB*	7.17 (1.92 to 26.76)	0.003
	*TEF*	4.59 (1.21 to 17.43)	0.025
	*HHEX*	6.13 (1.83 to 20.49)	0.003
	*SIK3*	3.26 (1.02 to 10.39)	0.046
	*IFITM1*	3.88 (1.04 to 14.48)	0.044
**Histologic Parameter**	**Genes**	**HR (95% CI)**	***p*-value**
Lymph node metastasis at diagnosis (yes vs. no)	*CDH13*	0.14 (0.02 to 0.77)	0.025
	*NELFB*	5.50 (1.16 to 26.14)	0.032
	*HHEX*	7.50 (1.61 to 34.95)	0.010
Predominant histologic subtype (ALM vs. other)	*DIPK2A*	0.04 (0.00 to 0.38)	0.005
	*NELFB*	0.04 (0.00 to 0.93)	0.044
	*TEF*	0.06 (0.01 to 0.38)	0.003
	*HHEX*	0.06 (0.01 to 0.38)	0.003
	*IFITM1*	10.82 (1.17 to 100.4)	0.036
Breslow thickness (>4 mm vs. 1.01–4 mm)	*CDH13*	0.12 (0.02 to 0.59)	0.009
Mitotic figures (≥10 vs. <4)	*CDH13*	0.19 (0.04 to 0.92)	0.040
	*TEF*	14.44 (1.56 to 133.6)	0.019
	*SIK3*	9.07 (1.72 to 47.67)	0.009
Ulceration (present vs. not identified)	*CDH13*	0.06 (0.01 to 0.56)	0.013
	*SIK3*	7.94 (1.60 to 39.42)	0.011
PNI (present vs. not identified)	*CDH13*	0.07 (0.01 to 0.64)	0.019

Abbreviations: CI, confidence interval; HR, hazard ratio; PNI, perineural invasion. * β score comparisons were hypermethylated versus hypomethylated.

## Supplementary Materials

The following are available online at https://www.mdpi.com/2072-6694/11/12/2031/s1, Supplementary Methods, Figure S1: (A) Unsupervised clustering with the top 50 differentially methylated positions in NALM, PCM, and AN; (B) Multidimensional parametric analysis of NALM, PCM, and AN, Figure S2: (A) MAPK signaling pathway shows enrichment in melanomas versus AN. Y axis, different gene sets with significant overlap with the probe sets/genes. X axis, ratio of the number of studied genes to the total number of genes included in the particular gene set (black dots). The dot sizes are proportional to the number of overlapping genes. The dot colors show the adjusted *p* value for false discovery rate (*p* < 0.01); (B) MAPK signaling pathway shows enrichment in NALM versus PALM, Figure S3: (A) Pathway analysis, MALM versus PALM. Note the very significant enrichment for MAPK signaling pathway gene sets in all comparisons except MALM versus PALM, where the MAPK pathway is preceded by the human papillomavirus (HPV) infection pathway. These findings highlight the importance of the inflammatory tumor microenvironment, which encompasses many genes in the HPV infection pathway in metastatic versus primary ALM; (B) Pathway analysis, PALM+NALM versus MALM, Figure S4: OS by (A) HHEX group, (B) NELFB/COBRA1 group, and (C) CDH13 group and (D) DSS by CDH13 group., Figure S5: Top differentially methylated probes common to the PALM versus MALM and PALM+NALM versus MALM analyses., Table S1: Pathway analysis. First sheet, NALM v PALM; second sheet, MALM v PALM+NALM; third sheet, MALM v PALM; fourth sheet, melanoma v nevus., Table S2: Significant promotor-associated differentially methylated probes for all comparisons. First sheet, NALM v PALM; second sheet, MALM v PALM+NALM; third sheet, MALM v PALM; fourth sheet, melanoma v nevus. Table S3: Beta scores for all analyses.
