# Supplementary material for: Aberrant DNA Methylation Predicts Melanoma-Specific Survival in Patients with Acral Melanoma
Source: Cancers (Basel). 2019 Dec 16;11(12):2031. doi: 10.3390/cancers11122031 (PMC6966546; doi:10.3390/cancers11122031)
Supplement: Supplementary file 1 [file cancers-11-02031-s001.zip › Supplementary Methods.docx]

**Supplementary Methods**

**MaterialS and methods**

**DNA Isolation, Methylation Analysis, and Functional Genomic Pathway Analysis**

DNA isolation, methylation analysis, and functional genomic pathway analysis were performed as described in our previous publication [54]. The methods are described in brief below:

**DNA Isolation**

Microdissection was performed on 10-μm-thick formalin-fixed, paraffin-embedded sections on the areas of greatest tumor cell density (at least 70% of the material analyzed). DNA extraction (Thermo Fisher) followed by bisulfite conversion (Zymo Research, Irvine, CA) was performed per the manufacturer’s instructions. Extracted DNA was hybridized on the bead chips provided by the manufacturer (Illumina, San Diego, CA).

**Methylation Analysis**

The Infinium Methylation EPIC array (Illumina) was used to determine the DNA methylation status of 866,562 CpG sites, following the manufacturer’s instructions. The minfi R package was used to process and analyze the methylation data [55]. Using minfi, the probes were quantile normalized and background adjusted. The resulting set of samples and probes was used for analysis of differentially methylated probes (DMPs). The DMPs finder function in minfi was used to identify DMPs for three different comparisons: (1) PALM versus NALM, (2) PALM versus MALM (3), and PALM and NALM (PALM+NALM) versus MALM. Probes with statistically significant differences in methylation with a Benjamini–Hochberg false discovery rate *q* cutoff of *q* < 0.01 were considered most significant, and corresponding heatmaps are shown. β-values for all 866,562 CpG sites tested were defined as the ratio of fluorescence intensity of the methylated probe to overall fluorescence intensity of probes. β < 0.2 indicated hypomethylation (blue); β > 0.8 indicated hypermethylation (red). All statistical analysis and modeling was performed using the open-source software R (<https://www.r-project.org/>). All graphs and heatmaps were generated using the R package. Unsupervised hierarchical clustering was done with Euclidean measure for distance matrix and complete agglomeration method for clustering.

Three PALMs and four PCMs could not pass the quality-control test for the assay and hence were excluded from the analysis.

**Functional Genomic Pathway Analysis**

All probes included in the array were annotated using the HumanMethylation850 manifest provided by the manufacturer (MethylationEPIC_v-1-0_B4; Illumina). Genomic information, including DNA sequences and coordinates of gene-coding regions, was obtained from the University of California Santa Cruz Genome Browser database [56]. All probes covering promoters and enhancers of coding genes were filtered in and considered for the enrichment pathway network analysis. This strategy was adopted to limit nonspecific enrichment pathway results that may occur when all coding and noncoding genes are included. Using the R package, we ran in parallel Cluster profiler and the DAVID Bioinformatics Resources interrogating 5867 genes in order to detect differentially methylated genes [57-60].

Using differentially methylated probes between the 2 compared groups, we perform pathway analysis / enrichment analysis (similar to GSEA but using the methylation data, not the gene expression data) plugging in the genes corresponding to these probes between the 2 groups. The output for the pathway analysis ranks the most enriched pathways between the two groups by the p and q values, as well as by the number of genes from that pathway that were included in the analysis over the total number of genes described in the pathway.

**Statistical Analysis**

Categorical variables were assessed using Fisher’s exact test (2 groups) or its generalization (> 2 groups), while continuous variables were evaluated using Wilcoxon rank-sum test (2 groups) or Kruskal-Wallis test (> 2 groups). The Kaplan-Meier method was used to estimate OS and DSS. Differences in survival between groups were assessed using the log-rank test. Optimal cutoff points were determined for β scores for outcome measures and histologic parameters with at least 10 patients in each category using the web application Cutoff Finder [61]. Associations between optimal cutoff groups and OS and DSS were assessed by univariate Cox proportional hazards regression models, whereas associations between optimal cutoff groups and histologic parameters were evaluated by univariate logistic regression models. All statistical analyses were performed using SAS 9.4 for Windows (SAS Institute Inc., Cary, NC). All statistical tests used a significance level of 5%. No adjustments for multiple testing were made.

**References**

54. Jour, G.; Vasudevaraja, V.; Prieto, V.G.; Snuderl, M.; Torres-Cabala, C.A.; Al-Rohil, R.; et al. BCAT1 and miR-2504: novel methylome signature distinguishes spindle/desmoplastic melanoma from superficial malignant peripheral nerve sheath tumor. Mod Pathol. 2019,32,338-345.

55. Aryee, M.J.; Jaffe, A.E.; Corrada-Bravo, H.; Ladd-Acosta, C.; Feinberg, A.P.; Hansen, K.D.; et al. Minfi: a flexible and comprehensive Bioconductor package for the analysis of Infinium DNA methylation microarrays. Bioinformatics. 2014,30,1363–1369.

56. Raney, B.J.; Dreszer, T.R.; Barber, G.P.; Clawson, H.; Fujita, P.A.; Wang, T.; et al. Track data hubs enable visualization of user-defined genome-wide annotations on the UCSC Genome Browser. Bioinformatics. 2014.30,1003–1005.

57. Huang da, W.; Sherman, B.T.; Lempicki, R.A. Bioinformatics enrichment tools: paths toward the comprehensive functional analysis of large gene lists. Nucleic Acids Res. 2009,37,1–13.

58. Huang da, W.; Sherman, B.T.; Lempicki, R.A. Systematic and integrative analysis of large gene lists using DAVID bioinformatics resources. Nat Protoc. 2009,4,44–57.

59. Yu, G.; Wang, L.G.; Han, Y.; He, Q.Y. clusterProfiler: an R package for comparing biological themes among gene clusters. OMICS. 2012,16,284–287.

60. Beissbarth, T.; Speed, T.P. GOstat: find statistically overrepresented Gene Ontologies within a group of genes. Bioinformatics. 2004,20,1464–1465.

61. Budczies, J.; Klauschen, F.; Sinn, B.V.; Győrffy, B.; Schmitt, W.D.; Darb-Esfahani, S.; et al. Cutoff Finder: Comprehensive and Straightforward Web Application Enabling Rapid Biomarker Cutoff Optimization. PLoS ONE. 2012, 7, e51862.

**Supplementary figure legends**

**Supplementary Figure S1 (A)** Unsupervised clustering with the top 50 differentially methylated positions in NALM, PCM, and AN; (**B)** Multidimensional parametric analysis of NALM, PCM, and AN.

**Supplementary Figure S2 (A)** MAPK signaling pathway shows enrichment in melanomas versus AN. Y axis, different gene sets with significant overlap with the probe sets/genes. X axis, ratio of the number of studied genes to the total number of genes included in the particular gene set (black dots). The dot sizes are proportional to the number of overlapping genes. The dot colors show the adjusted p value for false discovery rate (p < 0.01); (**B)** MAPK signaling pathway shows enrichment in NALM versus PALM.

**Supplementary Figure S3 (A)** Pathway analysis, MALM versus PALM. Note the very significant enrichment for MAPK signaling pathway gene sets in all comparisons except MALM versus PALM, where the MAPK pathway is preceded by the human papillomavirus (HPV) infection pathway. These findings highlight the importance of the inflammatory tumor microenvironment, which encompasses many genes in the HPV infection pathway in metastatic versus primary ALM; (**B)** Pathway analysis, PALM+NALM versus MALM.

**Supplementary Figure S4**. OS by (A) *HHEX* group, (B) *NELFB*/*COBRA1* group, and (C) *CDH13* group and (D) DSS by *CDH13* group.

**Supplementary Figure S5**. Top differentially methylated probes common to the PALM versus MALM and PALM+NALM versus MALM analyses.

**Supplementary table legends**

**Supplementary Table 1.** Pathway analysis. First sheet, NALM v PALM; second sheet, MALM v PALM+NALM; third sheet, MALM v PALM; fourth sheet, melanoma v nevus.

**Supplementary Table 2.** Significant promotor-associated differentially methylated probes for all comparisons. First sheet, NALM v PALM; second sheet, MALM v PALM+NALM; third sheet, MALM v PALM; fourth sheet, melanoma v nevus.

**Supplementary Table 3.** Beta scores for all analyses.
