# Supplementary figures and images for: Aberrant DNA Methylation Predicts Melanoma-Specific Survival in Patients with Acral Melanoma
Source: Cancers (Basel). 2019 Dec 16;11(12):2031. doi: 10.3390/cancers11122031 (PMC6966546; doi:10.3390/cancers11122031)

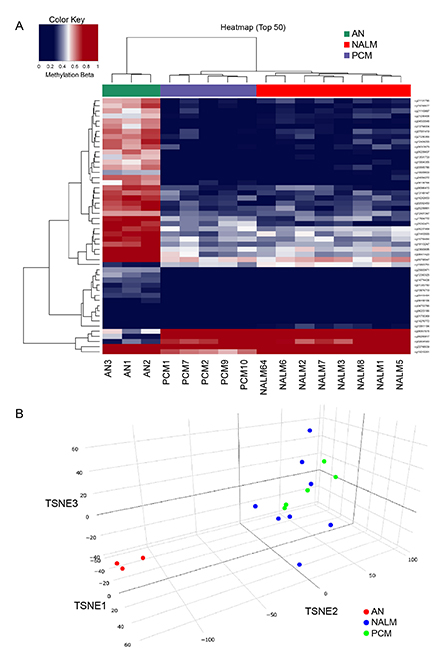

Supplement: Supplementary file 1 [file cancers-11-02031-s001.zip › Figure S1.tif]

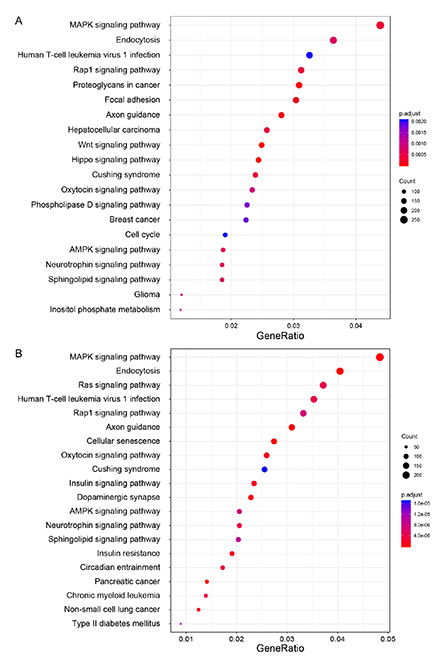

Supplement: Supplementary file 1 [file cancers-11-02031-s001.zip › Figure S2.tif]

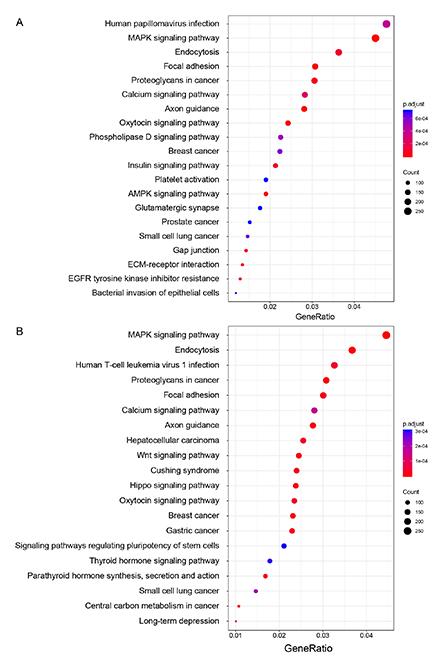

Supplement: Supplementary file 1 [file cancers-11-02031-s001.zip › Figure S3.tif]

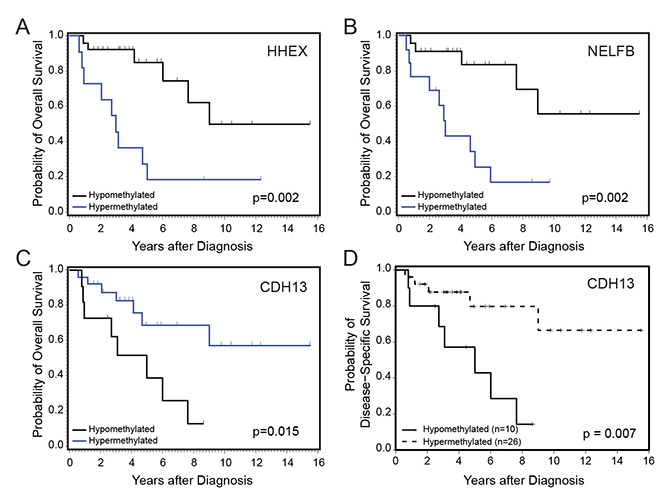

Supplement: Supplementary file 1 [file cancers-11-02031-s001.zip › Figure S4.tif]

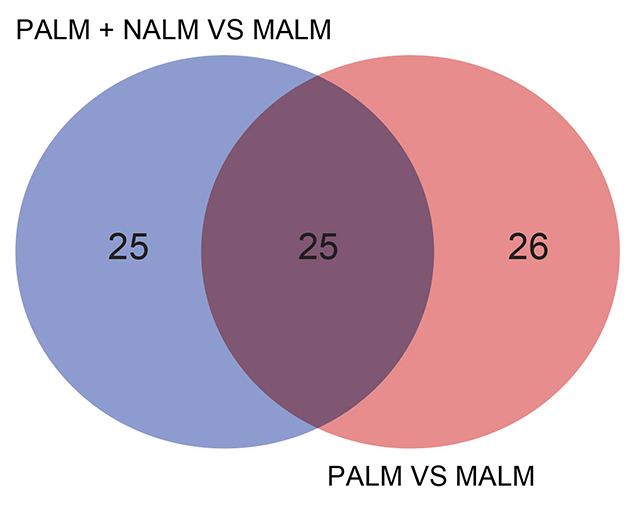

Supplement: Supplementary file 1 [file cancers-11-02031-s001.zip › Figure S5.tif]
